# Morphological variability of the palmaris longus muscle in human fetuses

**DOI:** 10.1007/s00276-018-2069-2

**Published:** 2018-07-18

**Authors:** Łukasz Olewnik, Anna Waśniewska, Michał Polguj, Michał Podgórski, Piotr Łabętowicz, Kacper Ruzik, Mirosław Topol

**Affiliations:** 1 Department of Normal and Clinical Anatomy, Interfaculty Chair of Anatomy and Histology, Medical University of Lodz, ul. Narutowicza 60, 90-136 Lodz, Poland; 2 Department of Diagnostic Imaging, Polish Mother’s Memorial Hospital Research Institute, Lodz, Poland; 3 Department of Angiology, Interfaculty Chair of Anatomy and Histology, Medical University of Lodz, Lodz, Poland

**Keywords:** Fetuses, Palmaris longus tendon, Palmaris longus muscle, Tendon grafts, Classification, Median nerve

## Abstract

**Purpose:**

The palmaris longus (PL) muscle is characterized by high-morphological variability. It is clinically important as it is routinely harvested for the reconstruction of other tendons. The study characterizes the morphology of the PL in human fetuses and creates a new classification based on its variations that would relate to the spectrum of morphological variability in adults.

**Methods:**

Eighty spontaneously aborted human foetuses (44 male, 36 female, 160 upper limbs), aged 18–38 weeks of gestation, were examined.

**Results:**

The palmaris longus muscle was present in 62.5% of fetuses. The absence was bilateral in 26.25%, and unilateral in 22.5%. Nine types of palmaris longus muscles were identified based on the morphology of its insertion (Types I–IX). All types originated on the medial epicondyle of the humerus. The most common type was Type I, which was characterized by insertion to the palmar aponeurosis (52%). The rarest types were Type VII and Type IX (1% each). Type VII was characterized by partial doubling of the muscle belly, which then turned into two separate tendons that inserted together into the palmar aponeurosis. Type IX was characterized by fusion with the flexor carpi ulnaris muscle.

**Conclusion:**

Our findings concerning morphological variability of the PL in fetuses present a new perspective on the understanding nature of the morphological variation of the PL muscle in adults.

**List of evidence:**

Basic Science Study.

## Introduction

The palmaris longus (PL) is a fusiform muscle, belonging to the superficial anterior compartment of the forearm. Its initial part originates together with other superficial forearm flexors from the medial epicondyle and is covered by the anterobrachial fascia [5, 14, 21, 25]. The short belly transforms into a long, thin tendon which lies medial to the flexor carpi radialis. It passes above the flexor retinaculum and fuses with the palmar aponeurosis [6, 21].

The PL is characterized by high-morphological variability including presence of an accessory muscle belly, occurrence of the reversed muscle, fusion with other muscles, the presence of an atypical tendon course, and inserts or bifurcated/multiple tendinous insertions [1, 5, 7, 15, 18, 20, 22, 25, 30]. In 1.5–63.9% of people (depending on the population) the PL may be absent unilaterally or bilaterally [8, 11, 21, 25, 28, 31]. Morphological variation of the PL is clinically significant because it is commonly used as a graft for tendons transfers. For the same reason, differences in the relation of the tendon to the median nerve should be studied in order not to mismatch the two [25]. Although the PL muscle does not have a vital impact on the normal functioning of the hand, the quality of the harvested tendon might depend on the muscle and tendon variant [4, 7, 10, 14, 15, 18, 20, 22, 25, 31–33].

Although there have been many studies on PL in adults, knowledge about the PL in human fetuses is scare. The morphological variability of the PL in adults might result from the variety of pathways involved in its development. However, in fetuses this muscle present in more forms than in adults. Hence, the purpose of this study is to characterize the morphological variability of the PL in human fetuses and to create a new classification that would serve as a link with the spectrum of morphological variability in adults.

## Materials and methods

Eighty spontaneously-aborted human fetuses (44 male, 36 female, 160 upper limbs), aged 18–38 weeks of gestation were examined. Permission for the study was given by the Local Bioethic Commission (agreement no. RNN/02/18/KE).

A dissection of the forearm and hand areas was performed by traditional techniques [24–27]. Firstly the PL muscle was exposed by the subcutaneous tissue and the antebrachial fascia. The morphology, together with the location and type of the insertion were evaluated. Morphometric and anthropometric measurements of the belly and tendon of the PL muscle were performed, and the distance between the midpoint of the interstyloid line (a line drawn between the styloid processes of the radius and the ulna) and the crossing of the median nerve and the PL muscle tendon were evaluated. An electronic digital caliper was used for all measurements (Mitutoyo Corporation, Kawasaki-shi, Kanagawa, Japan). Each measurement was carried out twice with an accuracy of up to 0.1 mm.

The morphometric measurements taken from different types of PL muscle were compared using the Statistica 12.0 software package (StatSoft, Cracow, Poland). The following tests were used: the Shapiro–Wilk test was used to determine the normality of the distribution; the chi^2^ test and the Mann–Whitney test were used to compare nominal and contentious variables between two groups, respectively; the Kruskal–Wallis ANOVA was used to compare contentious variables between more than two groups. The level of significance was 0.05, unless adjusted for multiple comparisons according to Bonferroni’s correction.

## Results

The “Results” section is divided into two parts. The first part concerns the morphology and absence of the PL, the second part describes the relationship between the tendon and the median nerve.

### Variation in PL morphology

The PL was present in 100 of 160 upper limbs (62.5%). The study recognized nine types of the PL muscle, based on variations in its form and insertion (Types I–IX). All types originated on the medial epicondyle of the humerus.

**Fig. 1:**
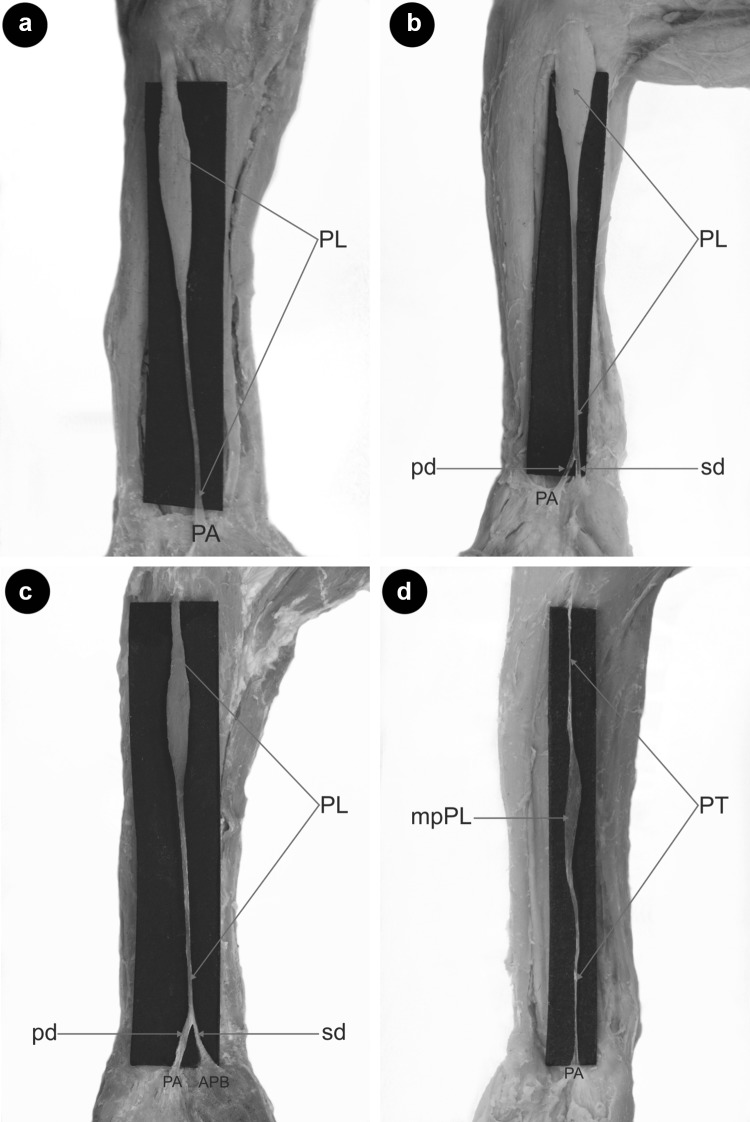
Type of palmaris longus muscle. **a** Type I palmaris longus muscle. Left forearm. *PL* palmaris longus muscle, *PA* palmar aponeurosis. **b** Type II palmaris longus muscle. Right forearm. *PL* palmaris longus muscle, *PA* palmar aponeurosis, *pd* palmar division, *sd* second division **c** Type III palmaris longus muscle. *PL* palmaris longus muscle, PA palmar division, *APB* abductor pollicis brevis, *pd* palmar division, *sd* second division. **d** Type IV palmaris longus muscle. *PT* palmaris tendon, *mpPL* muscular part palmaris longus muscle, *PA* palmar aponeurosis

Type I originated as a muscle belly on the medial humeral epicondyle. The muscular part turned into the tendon and inserted into the palmar aponeurosis. This type was found in 52 upper limbs (52%) (Fig. 1a).

Type II differed from Type I by the bifurcation of the distal tendon. Two components differed in size so that the ‘palmar division’ always predominated and inserted into the palmar aponeurosis (mean length 4.2 mm), while the ‘second division’ was auxiliary and inserted into the flexor retinaculum of the wrist (mean length 2.44 mm). The mean distance from the interstyloid line to the level of the tendon bifurcation point was 2.75 mm. This type was observed in 22 limbs (22%) (Fig. 1b).

Type III demonstrated a proximal attachment with the same morphology as Type I and II. The tendon was bifurcated; the ‘palmar division’ of the tendon always predominated and inserted into the palmar aponeurosis (mean length 3.70 mm), while the ‘second division’ fused with the abductor pollicis brevis (mean length 2.33 mm). The mean distance from the interstyloid line to the level of the tendon bifurcation was 1.54 mm. This type was found in 10 cases (10%) (Fig. 1c).

Type IV originated with a long, thin tendon from the medial epicondyle of the humerus, then the tendon gradually extended into an elongated muscle belly; slightly above the level of the interstyloid line, the muscle belly became a tendon once again, inserting into the palmar aponeurosis. This type was observed in six limbs (6%) (Fig. 1d).

**Fig. 2:**
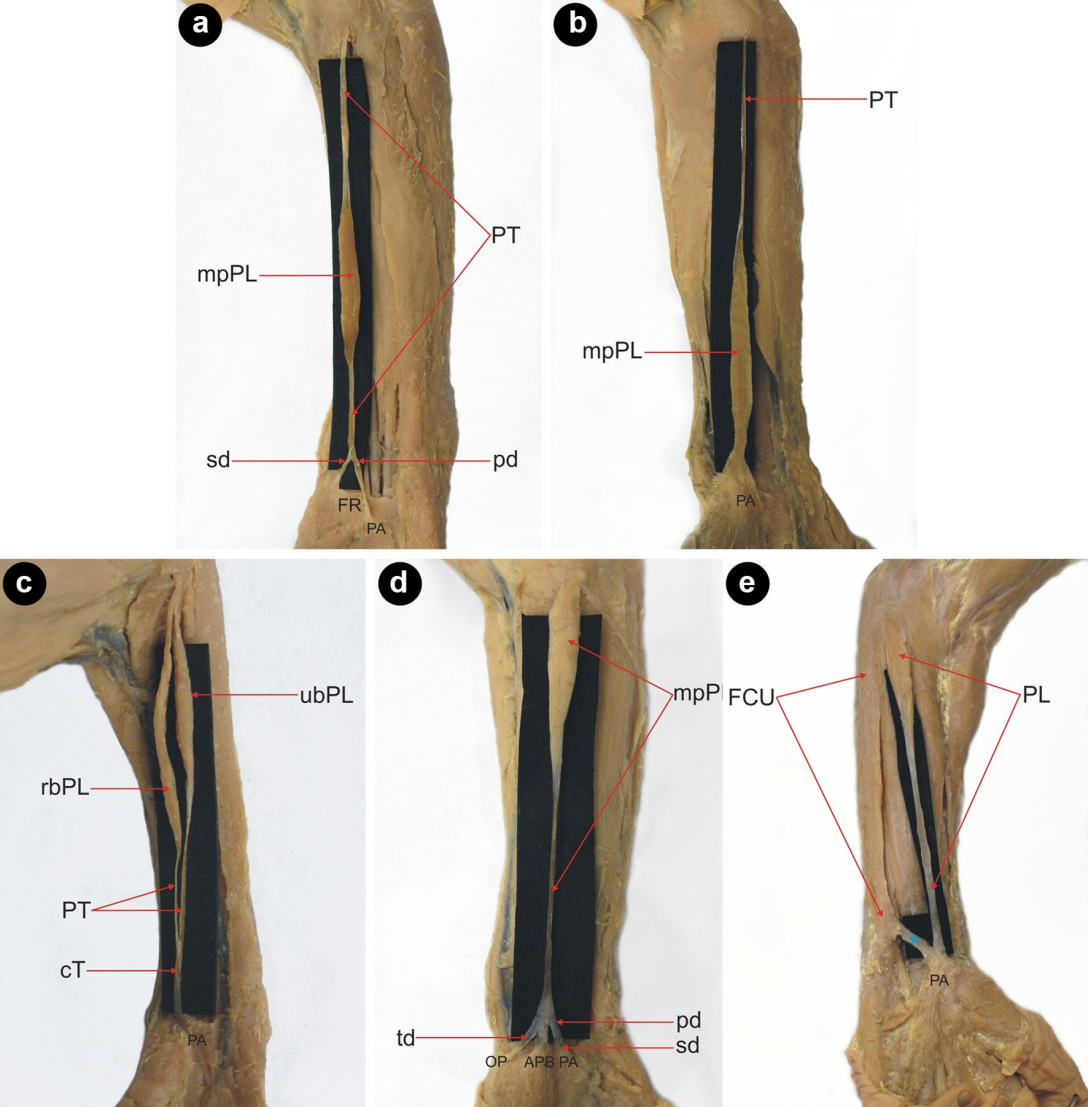
Type of palmaris longus muscle. **a** Type V palmaris longus muscle. *PT* palmaris tendon, *mpPL* muscular part palmaris longus muscle, *PA* palmar aponeurosis, *FR* flexor retinaculum, *pd* palmar division, *sd* second division. **b** Type VI (reversed) palmaris longus muscle. *PT* palmaris tendon, *mpPL* muscular part palmaris tendon, *PA* palmar aponeurosis. **c** Type VII palmaris longus muscle. *PT* palmaris tendon, *mpPL* muscular part palmaris longus muscle, *cT* communication tendon, *PA* palmar aponeurosis. **d** Type VIII palmaris longus muscle. *mpPL* muscular part palmaris longus muscle, *PA* palmar aponeurosis, *ABP* abductor pollicis brevis, *OP* opponens pollicis muscle, *pd* palmar division, *sd* second division, *td* third division. **e** Type IX palmaris longus muscle. *PL* palmaris longus muscle, *FCU* flexor carpi ulnaris, *PA* palmar aponeurosis × communication tendon between the palmaris tendon and flexor carpi ulnaris

Type V demonstrated the same morphology as Type IV; however, the distal tendon was bifurcated: the ‘palmar division’ predominated and inserted into the palmar aponeurosis (mean length 4.26 mm), while the ‘second division’ was auxiliary and inserted into the flexor retinaculum of the wrist (mean length 2.19 mm) The mean distance from the interstyloid line, between the styloid process, and to the level of the tendon bifurcation was 4.08 mm. This type occurred in three limbs (3%) (Fig. 2a).

Type VI—this type was reversed—with the proximal portion being tendinous and distal portion being muscular. It was found in two limbs (2%) (Fig. 2b).

Type VII—characterized by a partial doubling of the muscle belly, which then turned into two separate tendons (ulnar and radial). The length of the ulnar tendon was 7.47 mm, and the radial tendon 9.76 mm. The two tendons connect and inserted together into the palmar aponeurosis. This type was observed in one limb (1%) (Fig. 2c).

Type VIII—demonstrated a proximal attachment with the same morphology as Type I, II and III. The tendon was divided into three parts: the ‘palmar division’ of the tendon inserted into the palmar aponeurosis (mean length 4.72 mm), the ‘second division’ inserted into the abductor pollicis brevis (mean length 3.66 mm), and the ‘third division’ was auxiliary and inserted into the opponens pollicis muscle (mean length 3.07 mm). The mean distance from the interstyloid line to the level of the tendon bifurcation was 4.08 mm. This type was found in three limbs (3%) (Fig. 2d).

Type IX—the palmaris longus in the distal part passed above the palmar aponeurosis was connected as a ‘communication tendon and fused with the flexor carpi ulnaris muscle. This type was observed in one limb (1%) (Fig. 2e).

Morphometric measurements in different types of PL muscle are presented in Table 1.

**Table 1:** Relationship between types and morphometric measurements

Type	Forearm length (mm)	Forearm circumference (mm)	Width between proc. (%)	Thickness between proc. (%)	Belly length (mm)	Belly/forearm (mm)	Tendon length (mm)	Tendon/forearm (mm)	Thickness MT-junction (mm)	Width MT-junction (mm)	Thickness insertion point (mm)	Width insertion point (mm)
1 (52)	47.6 (10.6)	11.1 (4.1)	12.8 (3.9)	7.39 (3.1)	24.91 (7.70)	0.51 (0.11)	24.60 (7.97)	0.53 (0.12)	1.3 (0.5)	0.5 (0.2)	0.8 (0.4)	0.3 (0.1)
2 (22)	47.2 (10.3)	10.4 (2.8)	12.4 (2.9)	7.23 (2.2)	26.05 (6.88)	0.53 (0.09)	24.66 (5.28)	0.55 (0.07)	1.3 (0.4)	0.4 (0.2)	0.8 (0.3)	0.2 (0.1)
3 (10)	45.1 (11.9)	9.7 (3.1)	12.4 (3.9)	6.65 (2.2)	25.74 (6.36)	0.50 (0.03)	22.61 (6.47)	0.58 (0.06)	1.0 (0.3)	0.3 (0.1)	0.6 (0.3)	0.2 (0.1)
4 (6)	41.0 (2.6)	7.6 (1.3)	10.2 (1.3)	5.44 (1.0)	28.38 (4.97)	0.38 (0.06)	15.59 (2.62)	0.69 (0.11)			1.7	0.5
5 (3)	48.0 (5.0)	11.5 (0.5)	12.9 (1.1)	6.96 (0.9)	30.07 (2.63)	0.44 (0.09)	20.96(3.01)	0.63 (0.03)	1.3 (0.6)	1.0	1.5 (0.1)	0.7 (0.4)
6 (2)	45.7 (5.0)	10.2 (2.4)	11.9 (1.4)	6.49 (0.4)	20.41 (7.19)	0.59 (0.14)	26.68 (3.58)	0.44 (0.11)	1.1	0.4 (0.3)	1.6 (0.6)	0.7 (0.7)
7 (1)	34.3	4.2	5.2	3.2								
8 (3)	44.1 (6.6)	10.6 (2.9)	11.8 (1.8)	7.2 (1.5)	25.83 (1.09)	0.54 (0.04)	23.70 (4.01)	0.59 (0.09)	1.3 (0.2)	0.4	2.0	0.4
9 (1)	70.3	13.0	17.5	11.3	36.68	0.43	30.43	0.52	1.3	0.4 (0.1)	1.2 (0.5)	0.3 (0.2)
*p* value	0.3518	0.2249	1.000	1.000	1.000	1.000	1.000		1.000	1.000	1.000	1.000

Values are given as follows: number (%), millimeters (mm)

The PL was absent in 60 of 160 upper limbs (37.5%). The absence was bilateral in 21 of 80 fetuses (26.25%), and unilateral in 18 fetuses (22.5%). The bilateral absence was more common in males (13 cases; 16.25%) than in females (eight cases; 10%); however, this difference was not significant (*p* = 0.6275). Unilateral absence was observed in upper limbs in nine males (11.25%) and nine females (11.25%) (*p* = 0.5786). Unilateral absence was observed on six right (66.7%) and three left limbs (33.3%) in males (*p* = 0.1021), and on seven left (77.8%) and two right limbs (22.2%) in females (*p* = 0.7658).

### Relationship between the tendon of the PL and the MN

In this part, each type of palmaris longus tendon was checked for its relationship to the MN.

**Fig. 3:**
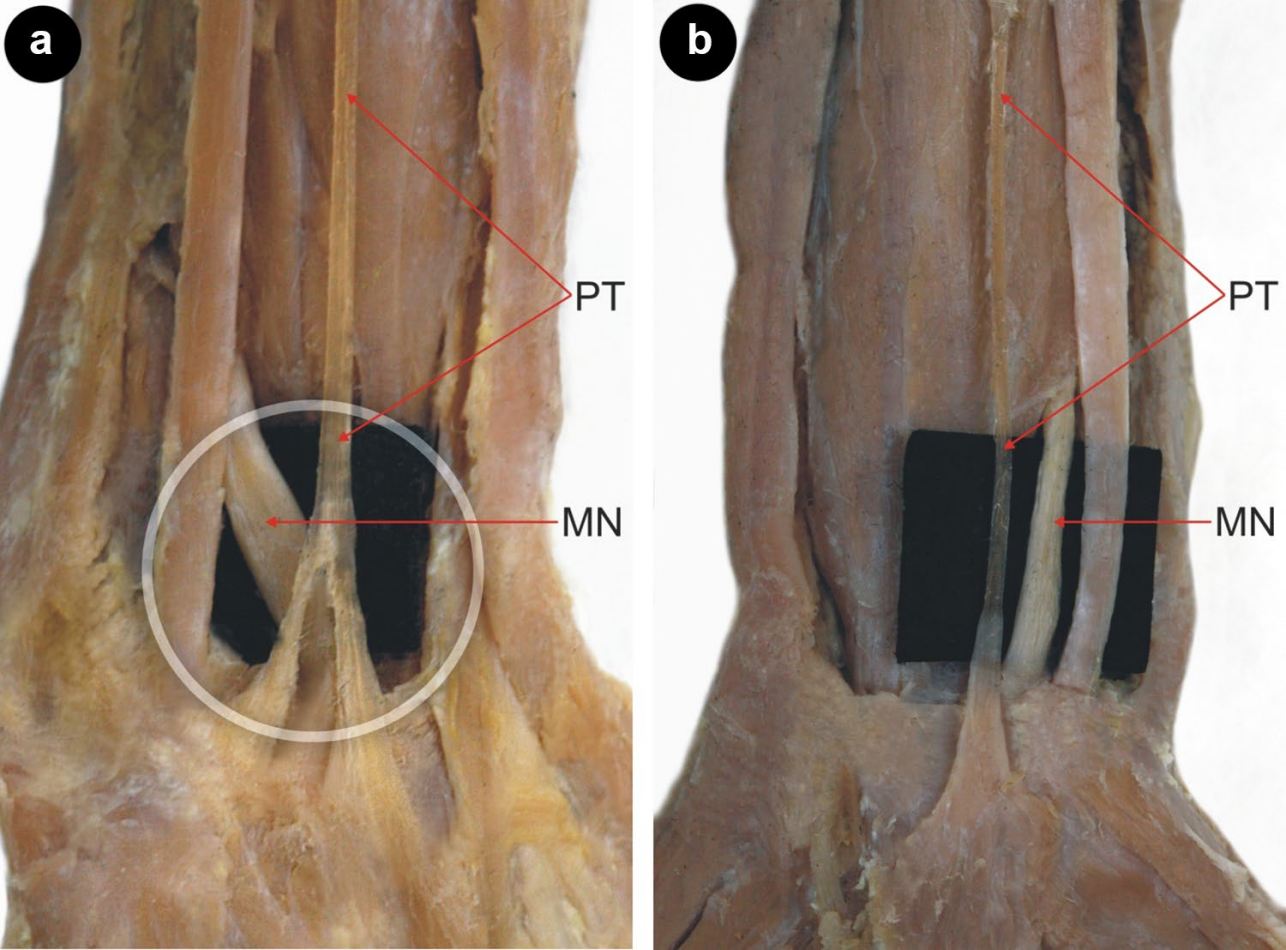
Relation between the palmaris longus tendon and median nerve. **a** At the crossing point between the median nerve and the tendon of the palmaris longus muscle. **b** No crossing between the median nerve and the tendon of the palmaris longus muscle

In type IV, none of tendons crossed the MN (Fig. 3b), whereas in Type II, III, VI, VII, VIII and IX all tendons crossed the MN (Fig. 3a). In type I and V, this was variable. In cases where the tendon crossed the MN, the distance from the midportion of the interstyloid line to the point where the tendon crosses the MN was the smallest in Type VII (2.63 mm) and the greatest in Type VIII (5.62 mm). Differences between types are given in Table 2.

**Table 2:** Relationship between the types of the PL and the MN

Types	Number of cases crossing the MN	Number of cases not crossing the MN	Mean distance from the midportion of the interstyloid line to the point where the tendon crossed the MN (mm)
I	47	5	4.21
II	22	–	5.61
III	10	–	3.90
IV	–	6	–
V	2	1	4.26
VI	2	–	4.28
VII	1	–	2.63
VIII	3	–	5.62
IX	1	–	4.36
*p* value	–	–	0.1234

In almost all types, both the mean width and mean thickness of the MN were greater than the mean width and mean thickness of the PL tendon at the cross point, except for Type VII, where the width of the PL tendon was greater than the width of the MN (Table 3).

**Table 3:** Mean width and thickness at the cross point between palmaris tendon and median nerve

Types of PL	WCMN (mm)	TCMN (mm)	WCP (mm)	TCP (mm)
I	0.811	0.244	1.524	0.772
II	0.750	0.234	1.444	0.620
III	0.785	0.189	1.302	0.585
IV	–	–	–	–
V	0.723	0.420	1.625	0.790
VI	1.120	0.575	1.710	0.660
VII	0.710	0.400	0.630	0.480
VIII	1.470	0.263	1.790	1.127
IX	1.150	0.310	1.330	1.010
*p* value	0.2993	0.1353	0.2987	0.1305

*WCMN* mean width of the tendon where the tendon crosses the median nerve, *TCMN* mean thickness of the tendon where the tendon crosses the median nerve, *WCP* mean width of the median nerve at the crosses point, *TCP* mean thickness of the median nerve at the crosses point

## Discussion

The PL muscle, like the plantaris muscle, is one of the most variable muscles in human body [1, 3–7, 9, 21–23, 25–27, 29, 32–34]. This is due to the rudimental function of this structures. More specifically, the PL muscle is well developed in species with a significantly higher ratio of upper limb weight to body weight. As this ratio is quite low in humans, i.e., the PL is less developed and its role in the functioning of the hand is reduced, it may present morphological variation [19].

With the development of the forelimb as a prehensile organ, the long flexor muscles of the forearm start to partially atrophy in a caudocranial direction. These muscles develop from the flexor mass, which is further divided into superficial and the deep layers. From the deep layer arise the flexor digitorum superficialis, flexor digitorum profundus and flexor pollicis longus, while the superficial layer gives rise to the pronator teres, flexor carpi radialis and ulnaris, and the PL [19]. Among vertebrates, the palmaris longus is restricted to mammals and is most well developed in species with a weight-bearing gait. The PL is always present in Orangutans, but is variably absent in chimpanzees and gorillas. Described as one of the muscles with the most anatomical variability, it is classified as a muscle in phylogenetic regression. Morphogenetically, its tendon and muscles are developed and regulated by the HOX gene [2, 13].

Anson et al. [3] and Reinman et al. [29] presented the first major study on the anatomical variations of the PL in adults. In 1600 upper extremities, the incidence of any kind of anomaly (except for the agenesis) was 45 in 540 consecutive arms, half of which concerned variations in position and form. In the remaining half, variations included additional slips and substitute structures (15 cases), duplication of the PL (four cases) and aberrancies of attachment (three times) [3, 29].

Although many original studies and case reports describe morphological variants of the PL muscle in adults, very few concerned fetuses [1, 17]. The present study distinguishes nine types of PL. Type I was the most common and was primary reported in both fetal studies [1, 17]. Type II, with a bifurcated distal tendon, was the second most common type, occurring in 22% of cases. Albay et al. [1] also report a similar type, but while the ‘second division’ inserted to the flexor retinaculum in our study, it passed deep to it. Kocabiyik et al. [17] did not report this type. Surprisingly, neither authors [1, 17] reported variants that gave additional slips to the abductor pollicis brevis (our third most common type—type III) or that were characterized by tendon–belly–tendon morphology (our types IV and V).

On the other hand, our type VI (reversed tendon–belly morphology), identified in 2% of cases, was also described by Albay et al. [1] and Kocabiyik et al. [17]. This variant is clinically significant because in adults it may predispose to median nerve compression by the muscular part of the PL [20, 22, 25]. A similar type to our Type VII (1%), characterized by partial doubling of the belly (muscular part), was described by Albay et al. [1], although in their case the muscular part was single and only the tendon was double. Type VIII (trifurcation of the tendon; 3% of cases) and Type IX (which was connected with flexor carpi ulnaris in distal part; 1% of cases) were not reported by Albay et al. [1] or Kocabiyik et al. [17]. Comparing the present results with those of previous studies, it can be concluded that many variations of the PL observed on the fetuses correspond to variants occurring in adults; however, Type V, VIII and IX did not [3, 4, 12, 14, 25, 29, 33].

In adults, the PL muscle may be completely absent in between 1.5 and 63.9% of examined subjects, depending on the population.

In the present study, the PL was found to be absent in 60 limbs (37.5%). We observed bilateral absence of the PL in 26.25% of fetuses, which is comparable with results of Albay et al. [1] (32.75%) and Kocabiyik et al. [17] (29.2%). More variation has been observed regarding unilateral absence, with it being reported in 22.5% of cases in the present study, 10.34% by Albay et al. [1] and 20.8% by Kacobiyik et al. [17]. For fetuses as well as for adults, the PL is more likely to be absent in female subjects and on the left side [1, 16, 29]. One study based on an Iranian population found the PL to be absent more often in men than in women, but this difference was not significant [23]. In the present study, male and female subjects presented the same rate of unilateral absence, although it was more common for the PL to be absent on the right side in males, but on the left in females.

Due to the fact that the PL is very often used for transplants, attention should be paid to its relationship to the median nerve. One potential grafting complication is a tendon tear. The place where it tears is most likely to occur at the tendon split, this being the location where the MN crosses the tendon. Our previous study on adult identified a crossing of the MN with PL tendon in most types, except for the single case, where a fusion was observed between the palmaris longus muscle and the flexor carpi ulnaris muscle [25]. At the cross point, it was observed that the MN was wider and thicker than the PL tendon [25]; a similar observation was made in the present study for all types apart from Type VII, and no crossing was observed for Type IV. In addition, five examples of Type I and one case of Type V did not cross the MN. In our previous studies conducted on adult humans, we observed a crossing point between the PL and MN in all types except the type that was a fusion with flexor carpi ulnaris [25]. The present study proves that in Type IV there was no place at the crossing point between the PL and MN, while in Types I and V there were cases of non-crossing. These are the first reports on the relationship between the PL and MN. Knowledge about the variability of these structures is essential for proper planning of surgical procedures.

This study has some limitations. Due to the great variability of the PL, the proposed classification is heterogeneous and it depends on several morphological details, such as the presence of accessory division, type of origin or insertion. Nevertheless, as it was created for research purposes, it has to be detailed rather than clinically simple and immediately usable in every day practice. Secondly, we are aware the presented types and their relation to the MN may change with gestation age and after birth. However, the variability of the PL muscle in the fetus is the best starting point for analyzing its variability in adult humans.

## Conclusion

Our findings shed new light on the morphological variation of the PL muscle present in fetuses. This may result in better understanding and interpretation of the variants of the PL present in adults. This is clinically significant because, despite its reduced role in the functioning of the upper limb, the PL tendon is common source of grafts for transplants.

## Abbreviations

APB: Abductor pollicis brevis
MN: Median nerve
PL: Palmaris longus
pd: Palmar division
sd: Second division
td: Third division
mpPL: Muscular part of the palmaris longus muscle
PA: Palmar aponeurosis
